# Prokaryotic Argonautes - variations on the RNA interference theme

**DOI:** 10.15698/mic2014.05.144

**Published:** 2014-04-15

**Authors:** John van der Oost, Daan C. Swarts, Matthijs M. Jore

**Affiliations:** 1 Laboratory of Microbiology, Wageningen University, Dreijenplein 10, 6703 HB Wageningen, Netherlands.; 2 Sir William Dunn School of Pathology, University of Oxford, South Parks Road, Oxford OX1 3RE, UK.

**Keywords:** Argonaute, RNAi, Bacteria, Archaea

## Abstract

The discovery of RNA interference (RNAi) has been a major scientific breakthrough. This RNA-guided RNA interference system plays a crucial role in a wide range of regulatory and defense mechanisms in eukaryotes. The key enzyme of the RNAi system is Argonaute (Ago), an endo-ribonuclease that uses a small RNA guide molecule to specifically target a complementary RNA transcript. Two functional classes of eukaryotic Ago have been described: catalytically active Ago that cleaves RNA targets complementary to its guide, and inactive Ago that uses its guide to bind target RNA to down-regulate translation efficiency. A recent comparative genomics study has revealed that Argonaute-like proteins are also encoded by prokaryotic genomes. Interestingly, there is a lot of variation among these prokaryotic Argonaute (pAgo) proteins with respect to domain architecture: some resemble the eukaryotic Ago (long pAgo) containing a complete or disrupted catalytic site, while others are truncated versions (short pAgo) that generally contain an incomplete catalytic site. Prokaryotic Agos with an incomplete catalytic site often co-occur with (predicted) nucleases. Based on this diversity, and on the fact that homologs of other RNAi-related protein components (such as Dicer nucleases) have never been identified in prokaryotes, it has been predicted that variations on the eukaryotic RNAi theme may occur in prokaryotes.

 Recent studies by us and the working group of Alexei Aravin have described molecular analyses of two distinct bacterial Argonautes, *Tt*Ago from *Thermus thermophilus *and *Rs*Ago from *Rhodobacter sphaeroides*. Like eukaryotic Argonautes, both *Tt*Ago and *Rs*Ago are long pAgos that are co-purified with oligonucleotide guides (13-25 nt and 15-19 nt, respectively), the majority of which are complementary to plasmids. Together with the observation that *Tt*Ago (and most likely also *Rs*Ago) target DNA, this has led to the conclusion that both pAgos play a role in host defense (Figure 1). However, apart from these similarities there are important functional differences between the two bacterial Argonaute proteins. The guides acquired by *Rs*Ago are mRNA-derived RNA oligonucleotides that target the template strand of plasmid genes. As *Rs*Ago lacks a catalytic site, it most likely requires a partner nuclease for target cleavage. In contrast, *Tt*Ago acquires DNA guides that allow targeting of AT-rich sequences of double-stranded plasmid DNA. Whereas the functional nuclease site of *Tt*Ago catalyzes nicking of a single targeted DNA strand, two *Tt*Agos loaded with overlapping complementary guides can generate double-stranded DNA breaks.

**Figure 1:**
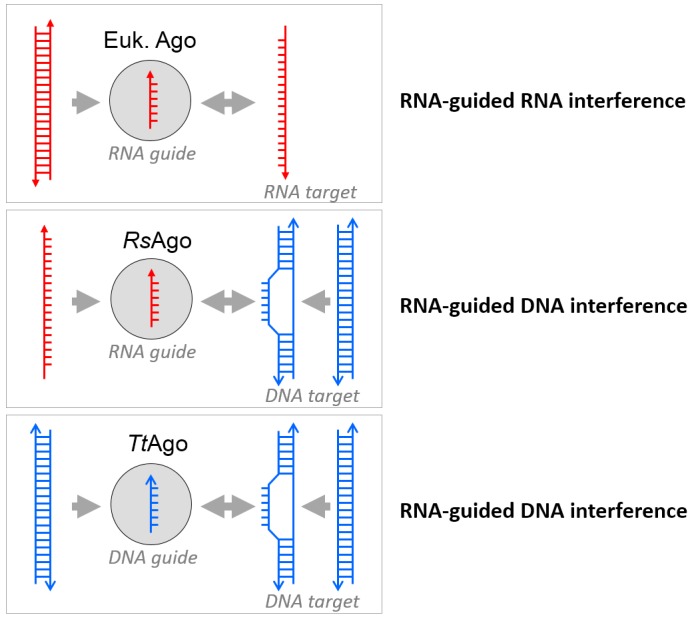
FIGURE 1: Prokaryotic variations of the eukaryotic RNAi theme. Eukaryotic Argonautes acquire short RNA guides to target complementary RNA molecules, resulting in silencing of expression of the corresponding gene; in case the Argonautes possess an intact active site, this will result in nucleolytic cleavage of the target RNA. Two types of prokaryotic Argonautes have recently been characterized. The RsAgo acquires guides from plasmid-derived mRNA, that allow binding of the complementary DNA strand of a plasmid; as RsAgo is inactive (*), it requires an additional nucleolytic enzyme for target cleavage. On the contrary, the active TtAgo acquires small DNA guides (mainly from plasmids) that allow for binding and cleavage of plasmid DNA strands.

These studies have revealed interesting variations on the eukaryotic RNAi theme. Several basic features of these two variant pAgos remain elusive, concerning mechanistic details of both guide acquisition and target interference. Moreover, apart from the two characterized prokaryotic Argonautes, many more pAgo variants exist, that may differ in functionality with respect to (i) guide preference (RNA/DNA), (ii) target specificity (RNA/DNA), and (iii) catalytic mechanism (nuclease activity). Apart from providing insights in the evolution of the prokaryotic Ago variants and their eukaryotic counterparts, future research will aim at revealing the molecular basis for the distinct functionality of these different pAgo variants. Moreover, gained insights will result in an interesting set of novel nucleases that may allow for dedicated genetic engineering.

